# A Community-Based, Cross-Sectional Study Assessing the Level of Awareness and Insight Related to Cardiovascular Diseases

**DOI:** 10.7759/cureus.15681

**Published:** 2021-06-16

**Authors:** Ali s Alghamdi, Muhanad S Alzahrani, Basel M Alsolami, Salman A Thabet, Basel s Alghamdi, Abdulhalim J Kinsara

**Affiliations:** 1 Internal Medicine, College of Medicine-Western Region, King Abdullah International Medical Research Center, King Saud Bin Abdulaziz University for Health Sciences, Ministry of National Guard Health Affairs, Jeddah, SAU; 2 Internal Medicine, College of Medicine-Western Region, King Saud Bin Abdulaziz University for Health Sciences, Jeddah, SAU; 3 Cardiology, College of Medicine-Western Region, King Abdullah International Medical Research Center, King Saud Bin Abdulaziz University for Health Sciences, Ministry of National Guard Health Affairs, Jeddah, SAU

**Keywords:** cardio vascular disease, saudi population, education, prevention, insight, awareness

## Abstract

Objective

The disease outcome had been shown to improve with improving patient knowledge. The study had two objectives, firstly to assess the level of knowledge about cardiovascular diseases (CVDs) in the general population, and secondly, to provide written educational material regarding the risk factors, major symptoms, and the prevention of CVDs.

Method

The target population was the residents living in the Western region of Saudi Arabia, aged 18 years and above. All were invited to participate voluntarily. A pre-structured questionnaire was designed to collect data related to age, gender, marital status, education level, occupation, lifestyle habits, and a history of heart diseases, as well as cardiac symptoms, and risk factors. The educational material was provided after the questionnaire.

Results

The majority of the participants were female (74.8%). The risk factors most frequently identified were lack of exercise, stress, and obesity. Chest pain was recognized as a major symptom (87.6%). Other symptoms included dyspnea, syncope, and excessive sweating. The level of knowledge regarding the risk factors for cardiovascular disease was poor. Only 18.5% were knowledgeable about the risk factors. The majority (60%) could identify the preventable factors, including smoking cessation (92.2%), a high level of cholesterol (88.6%), and hypertension (78.7%). The majority (83.7%) read the educational material and 99% reported that the lecture increased their knowledge about cardiovascular disease.

Conclusion

Although cardiovascular risk factors are common, there is a big gap in the knowledge in our population. Further, alarming symptoms that bring the patients to medical care are also deficient. A call for action at different levels is urgent. Simple educational material in a basic language and virtual education are useful and cheap tools that must be practiced wherever possible. Education is welcomed by the participants.

## Introduction

Cardiovascular diseases (CVDs) are the leading cause of death globally, with an estimated 17.9 million deaths annually. Four of five deaths are due to myocardial infarction and stroke, and one-third of these deaths occur prematurely in people under the age of 70 years [1]. The mean age for developing coronary artery disease (CAD) in the Middle East is 10 years younger than the mean age in Western countries [2]. A substantial increase in the prevalence of CVDs' risk factors in the Middle East has been reported [3], and the prevalence of CAD-related risk factors in Saudi patients with established CAD is concerning [4-5]. Improved knowledge about the disease may result in an earlier presentation to medical care and better patient outcomes. Individuals need to know and understand the implications of CVD, its symptoms, and risk factors to enable proactive minimizing of risk [6]. However, risk factors may vary between different regions and ethnicities [7,8]. A study from Eastern Saudi Arabia reported that only 3% were aware of a history of CVDs [9]. There is no literature related to the level of CVD knowledge in the Western region of Saudi Arabia. In addition, we included educational material to raise the level of CVD knowledge in the study population.

## Materials and methods

Study setting and participants: The design was a community-based cross-sectional study. Residents, residing in the Western region of Saudi Arabia, aged 18 years and older were invited to participate voluntarily. A pre-structured questionnaire was used to collect the data. The sample was calculated as 385 using the Raosoft Online Software (Raosoft, Inc., Seattle, WA, USA) [10]. The margin of error was 5%, with a 95% confidence level. The population size is approximately 10 million. Due to the possibility of incomplete responses, the sample was increased by 15% to 443.

Study variables: The questionnaire was previously validated [9, 11]. The online questionnaire included `six sections. The first section gathered demographic characteristics. The second section contained items exploring medical illnesses known to result in CVD: diabetes, hypertension, hypertriglyceridemia, hypercholesterolemia, smoking, and medication history. The third section focused on the risk factors for CVD, and the fourth section assessed the participants’ level of knowledge of CVD symptoms. The fifth section measured the level of knowledge regarding normal levels required to prevent CVD incidence for HbA1C, blood pressure and cholesterol, and smoking cessation). The last section contained educational material developed by the authors, followed by two questions to assess the benefit, and the perceived increase in the level of knowledge.

Statistical methods and ethical considerations: The categorical variables are presented as frequency and percentage, and the continuous variables as mean ± SD. The statistical tests used included a non-parametric Mann-Whitney U test to compare the differences between two independent groups, such as gender, should the dependent variable be ordinal or continuous, but not normally distributed. A non-parametric Kruskal-Wallis test was used for variables categorized in more than two groups, instead of the ANOVA. A non-parametric, related-samples Friedman test was used for variables categorized into more than two related groups. The knowledge levels were categorized as excellent (90 and above), very good (80-89), good (70-79), acceptable (60-69), and weak (below 60). The analysis was done with SPSS version 25. The study was approved by the Institutional Review Board of King Abdullah International Medical Research Center, SP20/348/J. The participants signed a consent form, indicating that participation was voluntary. No names or personal data were collected. The data were saved in a workplace computer to maintain confidentiality.

## Results

The study included 460 participants. The majority (74.8%) were female, 94.3% were Saudi, and 51.1% were married. The distribution of the age categories were 15.3% <20s, 32.4% 20-29 years; 9.6% 30-39 years, 23.3% 40-49 years, and 19.4% 50 years and above. The majority (69.7%) were university graduates, with 24.6% secondary school graduates (24.6%). Less than half (41.1%) were employed. The highest proportion (37%) had a normal weight, with a mean BMI of 25.9 ± 6.5 kg/m². A quarter (25.2%) smoked, with a mean of 9.6 ± 8.8 years. The most frequent comorbidities were hypercholesterolemia (15.4%), hypertension (11.7%), diabetes (11.5%), and a history of CVD (7%) (Table 1).

**Table 1:** Demographic characteristics of the sample.

	Characteristics	Count (%)
Age categories (years)	Below 20	70 (15.3)
	20-29	149 (32.4)
	30-39	44 (9.6)
	40-49	107 (23.3)
	50+	89 (19.4)
Level of education	Primary	6 (1.3)
	Intermediate	10 (2.2)
	Secondary	113 (24.6)
	University	321 (69.7)
	Diploma	4 (0.9)
	Masters	3 (0.7)
	Other	3 (0.7)
Income	Less than 5,000 SAR	225 (48.9)
	5,000–10,000 SAR	84 (18.3)
	10,001–15,000 SAR	74 (16.1)
	More than 15,000 SAR	77 (16.7)
BMI (kg/m²)	Mean ± SD	25.9 ± 6.5
	Underweight	54 (11.8)
	Normal	170 (37)
	Overweight	126 (27.5)
	Obese	109 (23.7)

There was a significant association between gender and CVD, with female dominance (p-value = 0.037). In terms of marital status, singles were more likely to have CVD (p-value = 0.013). Regarding age, there was a statistically significant association between the older age categories and CVD (p-value = 0.001). There was also a significant association between smoking and CVD (p-value =< 0.001) (Table 2).

**Table 2:** Age and sex association with cardiovascular disease. * - Significant ** - Extremely significant

	Characteristics	Count (%)	P-value
Gender	Male	13 (40.6)	0.037*
Age categories	Below 20	4 (12.5)	<0.001**
	20-29	5 (15.6)	
	30-39	0 (0)	
	40-49	4 (12.5)	
	50+	19 (39.4)	

The majority (80%) indicated lack of exercise, stress, and obesity as the most important risk factors. The mean overall knowledge score was 66.5 ± 23.8%. The majority (87.6%) listed chest pain or discomfort as symptoms, and the overall CVD knowledge score was 55.1 ± 17.5%. Regarding preventive methods, the majority (92.2%) considered smoking cessation as essential, a normal cholesterol level (88.3%), normal blood pressure (78.7%), and 75.9% a normal HbA1C level <7 (Tables 3 and 4).

**Table 3:** The sample’s level of awareness and insight related to cardiovascular disease symptoms.

Factors	Count (%)
Chest pain or discomfort	403 (87.6)
Cheat pain that radiates to the left arm	397 (86.3)
Shortness of breath	386 (83.9)
Feeling weak, light-headed, or faint	371 (80.7)
Pain or numbness in the arm	357 (77.6)
Loss of consciousness	332 (72.2)
Excessive sweating	247 (53.7)
The pain increased with exertion	244 (53)
Pain in the neck, jaw, or back	206 (44.8)
Fast heartbeats after high-intensity exercises	178 (38.7)
Pain relieved with rest or nitroglycerin	153 (33.3)
Abdominal pain	44 (9.6)
Overall knowledge (Mean ± SD) in %	55.1 ± 17.5

**Table 4:** Degree of awareness of different risk factors contributing to cardiovascular disease. LDL: Low-density lipoprotein.

Factors	Count (%)
Lack of exercise	416 (90.4)
Stress	392 (85.2)
Obesity	379 (82.4)
Hypertension	354 (77)
Current/Recent smoker (<1 year)	346 (75.2)
Family history of cardiovascular diseases	339 (73.7)
Unhealthy diet	331 (72)
Previous heart attack	324 (70.4)
High level of LDL Cholesterol	365 (79.3)
Smoking increases the risk of cardiovascular disease	311 (67.6)
Age	299 (65)
Cerebrovascular disease	270 (58.7)
Diabetes	259 (56.3)
Chronic kidney disease	194 (42.2)
Overall knowledge (Mean ± SD) in %	66.5 ± 23.8

The level of knowledge was classified into five groups depending on the correct responses: excellent (90 and above), very good (80-89), good (70-79), acceptable (60-69), and weak (below 60). The highest proportion (30.4%, n = 140) was in the weak knowledge group, 23.7% (n = 109) in the good knowledge group, 18.5% (n = 85) in the excellent knowledge group, 14.1 (n = 65) in the acceptable knowledge group, and 13.3% (n = 61) in the very good group (p-value = 0.001). The majority (58.5%, n = 269) had weak knowledge about the symptoms of cardiovascular disease, 20% good knowledge, and 19.3% acceptable knowledge. Only 2.3% (n = 10) were in the very good knowledge group. The sample’s level of knowledge regarding the factors that prevent CVD was mostly excellent (60%), good (23.9%), and weak (16.1%) (Table 5). 

**Table 5:** The assessment level of the sample towards risk factors, symptoms, and preventive factors of cardiovascular disease. ** - Extremely significant

Knowledge level	Excellent n (%)	Very good n (%)	Good n (%)	Acceptable n (%)	Weak n (%)	P-value
CVD risk factors	85 (18.5)	61 (13.3)	109 (23.7)	65 (14.1)	140 (30.4)	<0.001**
CVD symptoms	4 (0,9)	6 (1.3)	92 (20)	89 (19.3)	269 (58.5)	<0.001**
CVD preventive factors	276 (60)	0 (0)	110 (23.9)	0 (0)	74 (16.1)	<0.001**

A third (32%) of the sample was diagnosed with CVD: heart failure (28%), coronary heart [ASAA1] disease (25%), congenital heart disease (16%), arrhythmia (13%), and other diseases (19%). Of this group, only 65.6% were taking medication. The most frequently used cardiac medication were anticoagulation (47.6%), beta-blockers, and diuretics (38.1%), and only 4.8% were prescribed angiotensin-converting enzyme inhibitors (ACEIs).

## Discussion

This is the first study conducted in the Western region of Saudi Arabia, assessing the level of knowledge related to CVD risk factors, signs and symptoms, and preventive factors, in addition to providing educational material to the general population. The knowledge deficit that diabetes and chronic kidney disease were not considered as serious factors in causing CVD might explain the high prevalence and lack of control of these two risks. Similar findings were reported with a negative dietary intake being the highest risk factor, and only 12%, 26%, and 39% of the sample indicated that diabetes mellitus, smoking, and lack of physical activity were risk factors for CVD, respectively [2]. Although most participants were familiar with the most known CVD risk factors, the majority could not correctly identify all the risk factors. Similarly, many studies from developing African and Middle Eastern countries reported a lack of knowledge of CVD risk factors [3,12-14]. The majority (60%) of a sub-study with the Omani population had low knowledge scores related to the CVD risk factors [15]. In the United States, of 1,702 participants, half had adequate knowledge of CVD. The questionnaire asked the sample to identify 7 risk factors for CVD, and 37% of the group who responded correctly, identified all seven risk factors, with a mean of 4.9 risk factors [15].

In the current study, the majority (87.6%) indicated chest pain or discomfort as the most prominent symptom, higher than reported in studies from Kuwait and Saudi Arabia [2,16]. Being knowledgeable about CVD signs and symptoms is important, supporting early intervention and a better outcome. In Singapore, a delay in an intervention is mostly caused by the patient's inability to recognize the signs and symptoms of myocardial infarction [17]. It is concerning that the majority in the current study were not aware of the CVD symptoms. Similarly, a study revealed that 60% of the participants could not identify one symptom of myocardial infarction [18].

The current study also assessed the level of knowledge regarding the factors that prevent CVD, not frequently reported in the literature. Though almost all the participants indicated smoking cessation as an indispensable aspect of CVD prevention, a study reported that only half of the sample were aware that smoking cessation is a protective factor for CVD. This difference might be due to differences in the population [19].

The overall level of knowledge related to CVD risk factors was weak. The current study provides a unique assessment method for the level of knowledge related to preventative CVD factors. In addition, the study provided valuable educational material to reinforce knowledge about CVD and the risk factors. The majority of the sample (99%) indicated that the lecture was beneficial, and increased their knowledge (90.7%).

## Conclusions

The current study demonstrates a knowledge deficit regarding CVD risk factors, symptoms, management, and the association between each domain. Such knowledge will ensure early presentation to medical attention and improve the outcome. The educational material was beneficial and increased the level of knowledge, especially if social media was used. Additional educational campaigns are required to increase the level of knowledge about CVD. Further studies in different regions of Saudi Arabia are required to estimate the level of knowledge.
